# Neuroprotective Potential of Aromatic Herbs: Rosemary, Sage, and Lavender

**DOI:** 10.3389/fnins.2022.909833

**Published:** 2022-06-28

**Authors:** Arezoo Faridzadeh, Yasaman Salimi, Hamidreza Ghasemirad, Meraj Kargar, Ava Rashtchian, Golnaz Mahmoudvand, Mohammad Amin Karimi, Nasibeh Zerangian, Negar Jahani, Anahita Masoudi, Bahare Sadeghian Dastjerdi, Marieh Salavatizadeh, Hamidreza Sadeghsalehi, Niloofar Deravi

**Affiliations:** ^1^Department of Immunology and Allergy, School of Medicine, Mashhad University of Medical Sciences, Mashhad, Iran; ^2^Immunology Research Center, Mashhad University of Medical Sciences, Mashhad, Iran; ^3^Student Research Committee, Kermanshah University of Medical Sciences, Kermanshah, Iran; ^4^Student Research Committee, Shahid Sadoughi University of Medical Sciences, Yazd, Iran; ^5^Student Research Committee, Afzalipour Faculty of Medicine Kerman University of Medical Sciences, Kerman, Iran; ^6^Student Research Committee, School of Medicine, Shahid Beheshti University of Medical Sciences, Tehran, Iran; ^7^Student Research Committee, Lorestan University of Medical Sciences, Khorramabad, Iran; ^8^School of Medicine, Shahid Beheshti University of Medical Sciences, Tehran, Iran; ^9^School of Public Health, Iran University of Medical Sciences, Tehran, Iran; ^10^Student Research Committee, Faculty of Pharmacy, Mashhad University of Medical Sciences, Mashhad, Iran; ^11^Student Research Committee, Jahrom University of Medical Sciences, Jahrom, Iran; ^12^Student Research Committee, Department of Midwifery, Faculty of Nursing and Midwifery, Ahvaz Jundishapur University of Medical Sciences, Ahvaz, Iran; ^13^Department of Clinical Nutrition and Dietetics, Faculty of Nutrition and Food Technology, Shahid Beheshti University of Medical Sciences, Tehran, Iran; ^14^Department of Neuroscience, Faculty of Advanced Technologies in Medicine, Iran University of Medical Sciences, Tehran, Iran

**Keywords:** sage, lavender, rosemary, neurodegenerative, neurological disorders

## Abstract

Hundreds of millions of people around the world suffer from neurological disorders or have experienced them intermittently, which has significantly reduced their quality of life. The common treatments for neurological disorders are relatively expensive and may lead to a wide variety of side effects including sleep attacks, gastrointestinal side effects, blood pressure changes, etc. On the other hand, several herbal medications have attracted colossal popularity worldwide in the recent years due to their availability, affordable prices, and few side effects. Aromatic plants, sage (*Salvia officinalis*), lavender (*Lavandula angustifolia*), and rosemary (*Salvia Rosmarinus*) have already shown anxiolytics, anti-inflammatory, antioxidant, and neuroprotective effects. They have also shown potential in treating common neurological disorders, including Alzheimer's disease, Parkinson's disease, migraine, and cognitive disorders. This review summarizes the data on the neuroprotective potential of aromatic herbs, sage, lavender, and rosemary.

## Introduction

A neurological disorder is described as any condition that results in functional or structural damage to the nervous system. Neurological disorders account for the second main cause of death globally and the first main cause of disability as they typically cause cognitive impairment or sensorimotor dysfunction leading to reduced quality of daily life. Due to the high mortality and morbidity rate of neurological disorders, preventive and therapeutic strategies are crucial. The conventional medications administered for treating neurological disorders are associated with different adverse events; hence, the possible therapeutic effects of natural products on neurological conditions have been addressed by many researchers in the recent years (Ahmadi et al., 2022). A variety of herbal medications have gained the attraction of researchers in the last decade, due to their availability, lower price, and rare side effects (Abdel-Aziz et al., 2016).

Aromatic herbs such as sage (*Salvia officinalis*), rosemary (*Salvia Rosmarinus*), and lavender (*Lavandula angustifolia*) have shown promising neuroprotective effects in the recent studies (Kashani et al., 2011; Jamison, 2012; Jemia et al., 2013; Alvi et al., 2019; Mohseni et al., 2020; Caputo et al., 2021). *Salvia Rosmarinus* is an evergreen herb that belongs to the Lamiaceae family. *Salvia Rosmarinus* naturally grows in dry scrub and rocky areas in the Mediterranean regions of southern Europe to western Asia and has potential antibacterial, antifungal, antioxidant, and anti-inflammatory features (Leporini et al., 2020). The therapeutic effects of *Salvia Rosmarinus* on a variety of cognitive disorders such as Parkinson's disease, neuroblastoma, glioblastoma, and epilepsy have been suggested (Park et al., 2008; de Oliveira et al., 2016; Giacomelli et al., 2016; El Alaoui et al., 2017; Yildirim and Kitis, 2020). *Lavandula angustifolia* is a well-known aromatic herb in the Lamiaceae family. *Lavandula angustifolia* is native to the Mediterranean areas with antibacterial, antifungal, and antioxidant effects (Erland and Mahmoud, 2016). Neuroprotective properties of *Lavandula angustifolia* have been described in the literature. Dementia, glioblastoma, neuroblastoma, neurotoxicity, epilepsy, Parkinson's disease, and migraine have been found to respond to treatment with *Lavandula angustifolia* (Arzi et al., 2011; Sasannejad et al., 2012; Hancianu et al., 2013; Caputo et al., 2016, 2021; Nikolova et al., 2016; Chan et al., 2020). *Salvia officinalis* is a plant in the family of Labiatae/Lamiaceae. It is indigenous to the Middle East and Mediterranean regions. *Salvia officinalis* has been traditionally used for the treatment of a wide range of disorders such as seizures, ulcers, gout, rheumatism, and inflammation (Ghorbani and Esmaeilizadeh, 2017). The effects of *Salvia officinalis* on different neurological disorders such as cognitive deficits, glioblastoma, neurotoxicity, multiple sclerosis, and ischemic stroke have been reported (Iuvone et al., 2006; Kennedy et al., 2011; Seyedemadi et al., 2016; Li et al., 2018; Choukairi et al., 2020). This study aims to summarize the data on the neuroprotective potential of sage, lavender, and rosemary.

## Materials and Methods

The searched keywords for the present literature review consisted of sage (*Salvia officinalis*), Lavandula (*Lavender, Lavandula angustifolia*), rosemary (*Salvia Rosmarinus, Rosmarinus officinalis)*, dementia, glioblastoma, neurotoxicity, neuroinflammation, glioma, Alzheimer's disease, seizure, epilepsy, migraine, neuroblastoma, Parkinson's disease, neurodegenerative, oxidative stress, and neuroprotective. We searched for English articles in various online databases published in December 2021 (including Google Scholar, Web of Science, PubMed, Science Direct, Scopus, Embase, and ResearchGate). Hence, the extracted articles were reviewed.

### Rosemary

#### Cognitive Disorders

Cognitive disorders (such as amnesia, dementia, and delirium) are a class of mental health disorders mainly influencing learning, memory, and conception. Patients with cognitive disorders are not fully oriented to time and space (Festinger, 1957). Moss et al., investigated the effect of rosemary water on cognitive and cerebrovascular outcomes in healthy adults. Data from this study suggested the positive impacts of rosemary water on the improvement of mental activity. It was observed that the level of oxidized hemoglobin during cognitive function in the experimental group was significantly higher than the other groups, and the total data showed a positive and significant effect of rosemary juice. Therefore, rosmarinic acid compounds may simplify performance *via* cholinergic pathways (Moss et al., 2018). Yildirim et al. examined the effect of aromatic plants on cognitive disorders in old Adults. During this pre- and post-test study, 39 adults were given rosemary-lemon and lavender oil to smell for a week. Sleep quality and cognitive functions were assessed. The results showed that aromatherapy has a positive effect on cognitive functions in the elderly. In addition, it reduced the feeling of drowsiness during the day. Therefore, rosemary lemon oil is effective in controlling the quality of sleep and can improve cognitive disorders by controlling memory and calming effect on the sympathetic nerves (Yildirim and Kitis, 2020). In another study, Li et al. examined the effect of rosemary acid on cognitive impairment and hypoxia-ischemia, as well as myelin strengthening. In this study, ischemia-hypoxia-induced mice received 20 mg of rosemary acid intraperitoneally daily for 5 days. The results showed that rosemary acid could improve movement disorders, cognition, and spatial memory due to the effects of hypoxia-ischemia. Rosemary acid was also found to partially inhibit myelin degradation of corpus callosum neurons, and it can increase oligodendrocytes apoptosis inhibitors (Li et al., 2020). Song et al. showed that rosemary extract treatment in repetitive mild traumatic brain injury (TBI) of rats improved cognitive deficits through decreased neuronal degeneration and astrocytosis *via* reduced glial fibrillary acidic protein-positive cells, generation of reactive oxygen species (ROS), superoxide dismutase activation, catalase, glutathione peroxidase, protein levels of interleukin-1 (IL-1), IL-6, and tumor necrosis-alpha (TNF-α) in the hippocampus. Rosemary extract reduces the concentration of ROS and increases the activity of GPx, CAT, and SOD, reducing the number of degenerated neurons and improving cognitive deficits. As a result, rosemary extract may be a potential therapy for improving cognitive deficits in patients with repetitive mild TBI. Its mechanisms could be mediated by anti-inflammatory and antioxidative (Song et al., 2016). Several other studies also proved the positive effects of rosemary or its active compounds in cognitive disorders (Pengelly et al., 2012; Moss, 2017; Araki et al., 2020).

#### Results

**Figure 1:**
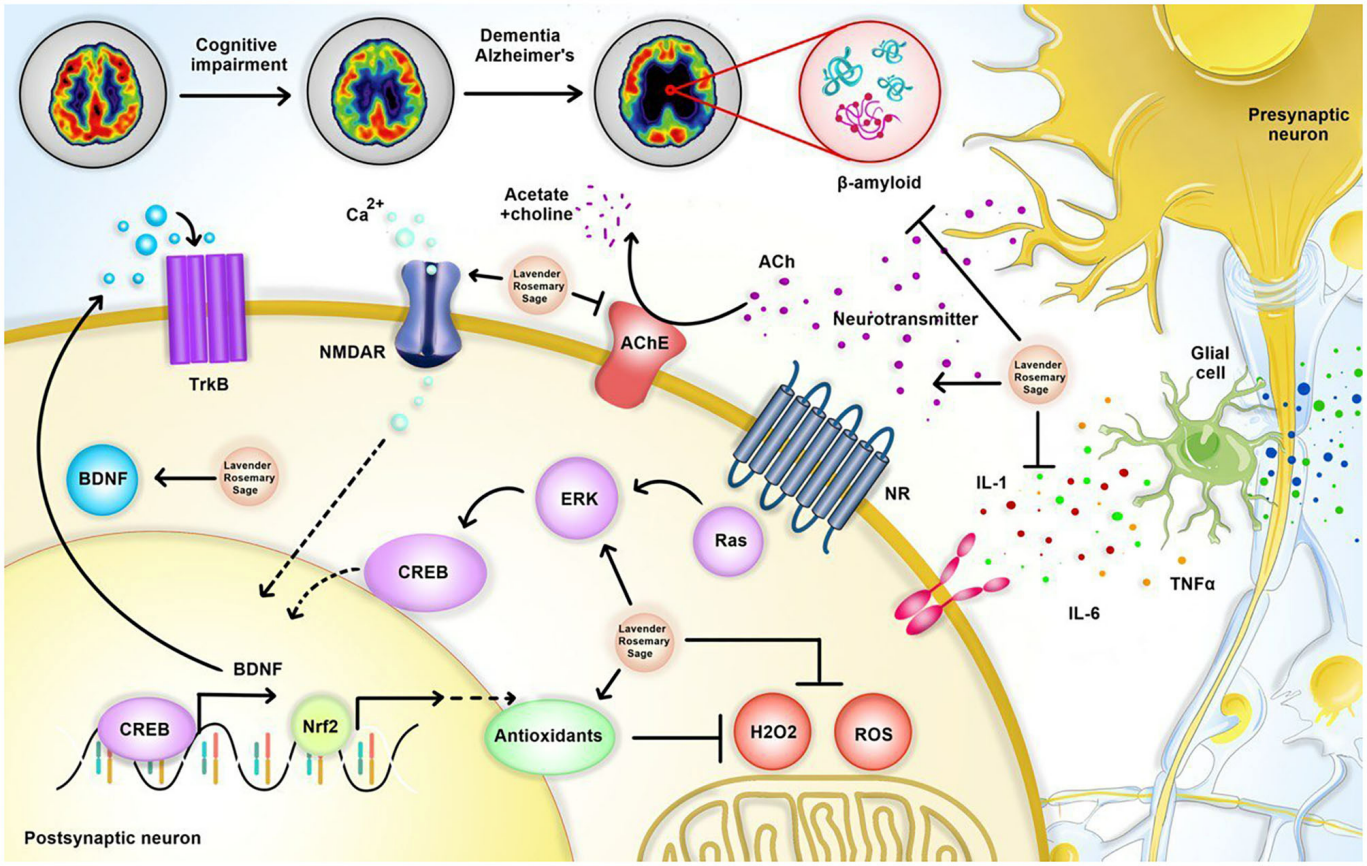
Neuroprotective effects of sage, lavender, and rosemary in neurological disorders. Sage, lavender, and rosemary exert neuroprotective effects mainly through increasing neurotransmitters and antioxidants, activating ERK/CREB/BDNF pathway, and inhibition of β-amyloid, pro-inflammatory cytokines, and Acetylcholine esterase (AChE) activity. BDNF, brain-derived neurotrophic factor; CREB, cyclic AMP response element-binding protein; NR, neurotransmitters receptor; ERK, extracellular signal-related kinase; TNF, tumor necrosis factor; TrkB, BDNF/NT-3 receptor.

In the following we will discuss the neurological effects of lavender, rosemary and sage in neurological disorders Figure 1 summarizes the neuroprotective effects of theses aromatic herbs.

#### Dementia

Dementia is a common disease caused by deterioration of cognitive function, especially as a result of aging. About 10 million new cases of dementia occur each year. Dementia is expected to be seen in 139 million people in 2050. Memory, comprehension, and judgment are affected by dementia. Alzheimer's disease (AD) is the most common cause of dementia, a continuous deterioration in thinking, behavioral, and social skills (Lane et al., 2018). The positive effects of rosemary on dementia and AD have been proved *in vivo* (Ozarowski et al., 2013; Lahouel et al., 2020). Ozarowski et al. demonstrated that *Rosmarinus officinalis* led to improvement in long-term memory and cognitive responses in rats, through the inhibition of acetylcholinesterase (AChE) activity and stimulation of butyrylcholinesterase (BuChE) in rat brain (Ozarowski et al., 2013). The inhibitory effect of rosemary on BuChE and AChE enzymes resulting in an increment in long-term memory has also been proved elsewhere (Karim et al., 2017). Javed Mirza et al. examined the highly neurogenic effects of *R. officinalis* and its active derivatives on AD and compared it to donepezil effects on AD. In this study, 10 groups of mice were examined. Half of them received amyloid-beta (Aβ)1-42 in the hippocampus and the other group received controlled injections. Each group of 5 was given ethanolic extracts of *R. officinalis*, ursolic acid, rosemary, and donepezil. The mice were then sacrificed and their hippocampal tissue was examined. Finally, it was observed that *R. officinalis* and its active components could inhibit neurotoxicity caused by AD. R. officinalis and its active compounds have antidepressant effects by regulating extracellular signal-regulated kinase-dependent mechanisms (ERK1/2) and enhance neurogenesis, synaptogenesis, and synapsin proteins. In addition to synapses, R. officinalis regulates the expression of synaptophysin (membrane protein in synaptic vesicles) and PSD-95 and the communication between neurons. Ursolic and rosmarinic acid also reduce Aβ-induced nerve damage by suppressing the oxidative pathway (Mirza et al., 2021). Lipton et al. examined the effect of carnosic acid (CA) extracted from *R. officinalis* and sage on AD. *In vitro*, the effects of CA on neurons exposed to oxidative stress were evaluated *in vivo*. Human amyloid protein-producing mice and triple transgenic mice were given CA by inhalation. Then, neurobehavioral and immunohistochemical tests were performed to assess its effects on AD such as learning. CA treated human amyloid protein-producing mice and also reduced the oxidative effect of stress on spinal neurons and increased dendritic and synaptic markers. CA stimulates the Keap1/Nrf2 (kelch-like ECH-associated protein 1/nuclear factor erythroid 2-related factor 2) pathway *in vitro* and *in vivo* and thus the production of phase 2 antioxidant enzymes and anti-inflammatory proteins (Lipton et al., 2016). The antioxidant effect of rosemary has also been studied elsewhere (Shalabalija et al., 2021). Yoshida et al. investigated the effect of CA on AD and reported that CA could induce a TACE / ADAM17 secretase and reduce amyloid 1-42 and 1-43 production as well (Yoshida et al., 2014).

Satou et al. reported the effect of rosemary essential oil on Alzheimer's disease induced by scopolamine in mice. They observed significant behavioral improvement in the rosemary group. Accordingly, 1,8-cineole and β-pinene from rosemary extract affected mice brains (Satou et al., 2018). Baron et al. reported the effect of rosemary extract on preventing AD by increasing glucose levels. Reduction of glucose uptake is seen in some neurodegenerative diseases such as AD. In an experiment on SH-SY5Y neuroblastoma cells, the result showed that there was an increase in the level of glucose uptake 2 h compared with the maximum insulin stimulation. This effect was observed with the upregulation of AMP-activated protein kinase (AMPK) (Baron et al., 2021). Jimbo et al. experimented the effects of aromatherapy with two aromatic herbs, including lemon and rosemary essential oils in the morning and orange and lavender in the evening, for the treatment of AD. A noticeable improvement in the Steen scale and Touch Panel-type Dementia was observed after aromatherapy. The laboratory tests revealed that there was no significant side effect for aromatherapy. Zarit's score indicated that caregivers did not affect the improvement in patients' scores (Jimbo et al., 2009). Furthermore, Okuda et al. investigated the effect of aromatherapy on improving cognitive abilities. In this study, several mice with AD were examined. They gave the mice lemon and rosemary oil every night. Lavender and orange oil were given during the day. The cognitive functions of mice were then assessed before and after the experiment. Both fetal Aβ and neutron factor levels were evaluated after the treatment. It was observed that the amount of abnormal Aβ and phosphorylated tau decreased in the group treated with aromatherapy and the amount of BDNF increased slightly, all of which indicated the effectiveness of aromatherapy in the treatment of diseases such as AD (Okuda et al., 2020). In another study by Leu et al., the impact of CA on Aβ-induced injury in SH-SY5Y neuroblastoma cells was investigated. CA pretreatment lessened the loss of cell viability caused by Aβ25–35. It also decreased the production of reactive oxygen species. CA had an important role in maintaining the potential of the mitochondrial membrane. CA caused autophagy by increasing the amount of light chain 3 (LC3)-II/I ratio and lessening SQSTM1(p62). CA also induced autophagy by activating AMP-activated protein kinase (AMPK). These results indicated the role of CA in the prevention of Alzheimer's disease (Liu et al., 2016).

#### Parkinson's Disease

Parkinson's disease (PD) is a brain disorder leading to stiffness, shaking, and trouble with balance, walking, and coordination. PD's symptoms generally begin gradually and often get worse over time. The progression of the disease leads to problems in talking and walking. PD is caused by a loss of nerve cells, responsible for producing dopamine, in the substantia nigra (Marino et al., 2020).

Carnosol is the main component of *R. officinalis*, which has powerful antioxidant and anti-inflammatory effects. Kim et al. investigated the protective effects of carnosol on the rotenone-induced neurotoxicity of cultured dopaminergic neuronal cell line (SN4741) (Kim et al., 2006). Along with it, they investigated the protective effects of carnosol on dieldrin-induced neurotoxicity in cultured dopaminergic neuronal cells (SN4741). The neuroprotective effect of carnosol is related to the inhibition of cell death in dopamine neuron cells and upregulation of tyrosine hydroxylase expression, which occurs *via* the Raf-MEK-ERK1/2 pathway (Raf-mitogen-activated protein kinase (MEK)-extracellular signal-regulated kinase (ERK)1/2 signaling pathway). Carnosol, as a potent antioxidant, could have therapeutic value in the improvement in PD symptoms and the dopamine system (Park et al., 2008). In another study, Chen et al. evaluated whether CA, a phenolic diterpene found in rosemary, can attenuate the adverse and neurotoxic effects of 6-hydroxydopamine on SH-SY5Y cells. 6-hydroxydopamine is a powerful neurotoxin that can injure dopaminergic neurons of animals and also cell models of PD. Their study indicated that Nrf2-induced synthesis of glutathione, leading to downregulation of p38 and c-Jun N-terminal kinase signaling pathways, is how CA attenuates apoptosis derived from 6-hydroxydopamine. Glutathione is an essential antioxidant that can be found in neurons and astrocytes. It is crucial in the cell protection against the toxicity of ROS, which has a significant role in the death of dopaminergic cells in PD. Therefore, CA might potentially be a candidate to protect against neurodegeneration seen in PD (Chen et al., 2012).

Park et al. investigated the possible neuroprotective effects that rosemary extract may have on apoptosis induced by H_2_O_2_ in human dopaminergic cells, SH-SY5Y. The results indicated that treatment with rosemary suppresses cytotoxicity caused by H_2_O_2_ in SH-SY5Y cells. In addition, rosemary effectively attenuates disruption of the mitochondrial membrane potential as well as H_2_O_2_-induced apoptotic cell death. The downregulation of Bcl-2 and upregulation of caspase-3, caspase-9, Bak, and Bax were also suppressed by this extract. Moreover, pretreatment with rosemary remarkably attenuated the downregulation of two genes in SH-SY5Y cells, including tyrosine hydroxylase and aromatic amino acid decarboxylase. Due to these results, rosemary may have a potential in protection of neurons against H_2_O_2_-induced injury. It also acts as a prophylaxis for various neurodegenerative diseases caused by apoptosis or oxidative stress, for instance, Parkinson's disease (Park et al., 2010).

#### Oxidative Stress

The brain is particularly vulnerable to oxidative stress. The involvement of reactive oxygen species (ROS) in the pathogenesis of many diseases of the central nervous system is widely established. The plasminogen activator system plays an important role in oxidative stress. Lavrentiadou et al. studied an experimental rat model of ROS-induced through intraperitoneal administration of CCl4, which led to induced oxidative stress and activation of the PA system in the brain of rats. The consumption of rosemary in rats affects the inhibition of PA activation that can be able to protect the rat's brain from neuronal damage (Lavrentiadou et al., 2013). Hamed et al., for the first time, explored the prophylaxis and therapeutic efficacy of *R. officinalis* extracts against chronic toxoplasmosis, which leads to delayed death in mice infected with the Toxoplasma gondii. Histopathological observation of brain and liver tissue of mice exhibited increased iNOS stain expression, which demonstrates antioxidant activities. Treatment with these extracts displayed a reduction in cyst burden and cyst viability through inhibition of parasitic BAG-1 gene expression (Hamed et al., 2021). Asl et al. showed that rosmarinic acid plays a crucial role in the elevation of protein nitric oxide, malondialdehyde, carbonylation, and significant reduction of total antioxidant capacity, glutathione, glutathione peroxidase, catalase, and superoxide dismutase in the radio frequency radiation-exposed rats' brain compared to the control group. Hence, it may protect against oxidative stress *via* ameliorative effects on the antioxidant activities in brain tissues (Asl et al., 2020). In another study, Satoh et al. found out that CA obtained from *R. officinalis* binds to specific Keap1 cysteine residues, leading to the activation of the Keap1/Nrf2 transcriptional pathway, causing protection of neurons against excitotoxicity and oxidative stress, in an *in vivo* and *in vitro* model (Satoh et al., 2008). The effect of CA on upregulating endogenous antioxidant enzymes through the Nrf2 pathway has also been evaluated elsewhere (Zhang et al., 2015). In another study, Posadas et al. hypothesized that the extract of rosemary could strengthen antioxidant defenses and enhance the antioxidant status of aged rats. Thus, they fed aged rats with two different concentrations of supercritical fluid rosemary extract (0.2 and 0.02%) for 12 weeks. The results indicated that rosemary diminished lipid peroxidation in brain tissues of rats consuming both doses. Moreover, supercritical fluid extract rosemary-treated rats showed a lower ROS level in their hippocampus, compared to the control rats. The level of catalase activities was also diminished in the rosemary-treated group. In conclusion, these observations suggested that rosemary supplements can improve the oxidative stress status of old rats (Posadas et al., 2009).

#### Neuroblastoma

Neuroblastoma is a malignant cancer, which develops in immature nerve cells called neuroblasts. Adrenal glands, chest, neck, and spinal cord are the most common places for neuroblastoma to begin. It is observed in children more than in adults and is the third most common cancer in children after brain and spinal cord tumors and leukemia (Riley et al., 2004).

Oliveira et al. reported the role of *R. officinalis* as a mitochondrial protector in Sh-Sy5y cells of human neuroblastoma. Decreased levels of markers of oxidative stress, inhibition of caspases 3 and 9, and cytochrome C (as the factors of mitochondrial membrane disruption) were observed after treatment with CA. Activation of erythroid 2-related factor 2 (Nrf2) can induce that the signaling pathway phosphoinositide-3-kinase (PI3K) by CA leads to upregulation of glutathione. So, anti-apoptotic and antioxidant effects are the reason of this mechanism (de Oliveira et al., 2016). In another study, Oliveira et al. found out that CA has an anti-inflammatory effect on SH-SY5Y cells by inhibiting NF-κB and acting on Nrf2/ heme oxygenase-1 pathway (de Oliveira et al., 2018). Oliveira et al. also reported similar results in another study (de Oliveira et al., 2017). Another study reported mechanisms of apoptosis activation in IMR-32 neuroblastoma cells induced by CA. CA caused a reduction in Bcl-2, a protein against apoptosis. Prevention of the activity of siRNA and caspase 3 was also observed (Tsai et al., 2011).

#### Glioblastoma

Glioblastoma multiforme (GBM) is the most common malignant tumor of the CNS system that happened in the brain or spinal cord. Necrotizing tissue and anaplastic cells are observed in GBM. It is more common in men over 60 years old. The prognosis of the tumor is poor (Thakkar et al., 2014).

Özdemir et al. claimed that human cytomegalovirus (CMV) is associated with GBM. Therefore, they investigated the effect of rosemary and acyclovir on glioblastoma cells. Rosemary reduces GBM survival. Rosemary also reduces the expression of survivin (an anti-apoptotic protein). Survivin is a caspase inhibitors 3 and 7 and has been shown to reduce survivin regulation related to cytotoxic conditions. MTT assay revealed a 57.2% reduction in GBM cells survival induced by rosemary. The combination of rosemary and acyclovir reduced the survival by 10% more. A 52.7% reduction in the viability of GBM was seen in the acyclovir group (Özdemir and Göktürk, 2019).

**Figure 2:**
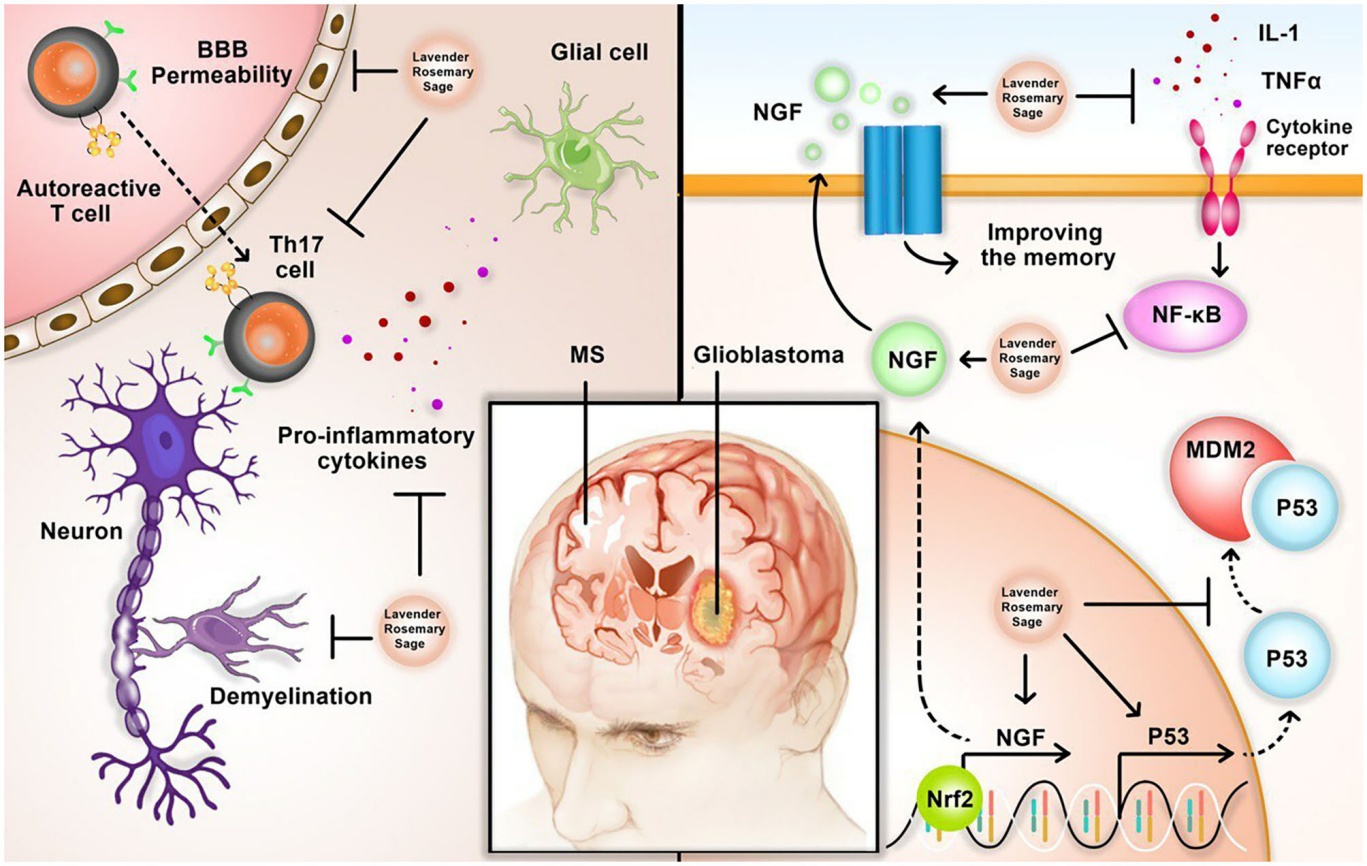
Neuroprotective effects of sage, lavender, and rosemary in Multiple sclerosis (MS) and glioblastoma. In MS, herbal medicine can inhibit Blood-Brain Barrier (BBB) permeability, T helper 17 (Th17) polarization, pro-inflammatory cytokines secretion, and axonal demyelination. In glioblastoma, herbal medicine can induce transcription of p53 and NGF, resulting in memory improvement. Sage, lavender, and rosemary can also inhibit nuclear factor κB (NF-κB), pro-inflammatory cytokines, and MDM2, which is an inhibitor of tumor suppressor p53.

In another study, Giacomellia et al. studied the effects of carnosol, found in rosemary, on U87MG GBM cells. P53 is a regulator of cell apoptosis. The expression of p53 is often impaired in glioblastoma. Carnosol acts as an apoptosis inducer by reactivating the p53 pathway in GBM cells. Also, carnosol, together with temozolomide, prevented GBM cells from proliferation (Giacomelli et al., 2016). Figure 2 summarizes the neuroprotective effects of lavender rosemary and sage in glioblastoma.

#### Glioma

One of the most dangerous tumors for the human body is glioma. The glial cells are the origins of gliomas. The incidence of glioma is about 6–10 per 100,000 people. Headache, nausea, memory loss, speech difficulties, and confusion are some of the common symptoms of glioma. Unfortunately, the prognosis is poor (Gore et al., 2021).

Huang et al. investigated the effects of ursolic acid as one of the active compounds of *R. officinalis*. The results showed that C6 glioma invasion induced by IL-1β or TNF-α was reduced by ursolic acid. They also found out that ursolic acid did not affect cell toxicity or proliferation of the host cells. It also reduced matrix metalloproteinase-9 (MMP-9) as the target gene of the transcription factor nuclear factor-kappaB (NF-kB). UA increased IkappaB-alpha (IkBα). Translocation of p65 was reduced by UA. Finally, the researchers concluded that UA from rosemary extracts is useful for the prevention of the invasion and metastasis of glioma (Huang et al., 2009).

#### Epilepsy

Epilepsy is defined as a condition of a patient who suffers from recurrent and spontaneous seizures. A seizure is an abnormal electrical discharge in the brain which ultimately causes a severe unconscious muscle spasm, which usually is associated with a decreased level of consciousness. The incident rate of seizures is more in men, infants below 12 months, and people above 65 years old (Hauser and Beghi, 2008; Huff and Fountain, 2011; Saito and Terayama, 2013). Each year 50 out of 100,000 people are diagnosed with epilepsy (9). Epilepsy is the third most common neurologic condition globally (Blair, 2012; Azimi et al., 2021).

Alaoui et al. studied the effects of rosemary and lavender on the neurological system by investigating the usage of these aromatic herbs in modulating T-type calcium channels (TTCCs). TTCCs affect sleep, neuroprotection, and sensory processes. Pain and epilepsy are also affected by TTCC. The researchers found out that rosmarinic acid, linalool (the active compounds of rosemary), and lavender can inhibit TTCC (Ca v3.2) current, respectively. Rosemary and lavender also induce a negative change in the steady-state inactivation of this current. It can be concluded that these two herbs play an important role in controlling epilepsy by the inhibition of TTCC (El Alaoui et al., 2017).

#### Migraine

Migraine induces a mixture of symptoms, most notably a throbbing, pulsing headache on one side of the head. Pain is sometimes expressed as pounding or throbbing. It can start as a dull ache that expands into pulsing pain that is light, moderate, or severe (Goadsby et al., 2002). BK Göksel et al. showed that rosemary is a complementary and alternative medicine (CAM) that can be useful in primary headache syndromes (Karakurum Göksel et al., 2014).

#### Neurotoxicity

Neurotoxicity is defined as a functional or structural disorder in central or peripheral neural system cells. This disorder is caused by different chemical or biological factors, and it can affect neurochemical, behavioral, neurophysiological, or anatomical aspects (Erinoff, 1995; Tilson et al., 1995; Slikker and Bowyer, 2005). The effect of rosemary on neurotoxicity has been evaluated. Balawi et al. showed that treatment with rosemary extracts improved acrylamide-induced neurotoxicity in all brain areas of albino rats (including the brain stem, striatum, cerebellum, cerebral cortex, hippocampus, and hypothalamus) (Balawi, 2010). Oliveira et al. investigated the role of CA in preventing neurotoxicity induced by methylglyoxal in SH-SY5Y neuroblastoma cells. Glycation is a process that leads to neurodegeneration in diseases such as Alzheimer's disease. Methylglyoxal is a powerful inducer of glycation end products. In this study, the authors expressed that CA in rosemary had a significant role in increasing anti-oxidative effects and acting against the outcomes of methylglyoxal. CA prevented the release of cytochrome C and loss of mitochondrial membrane polarity (MMP) and subsequently blocked the activation of proapoptotic caspase enzymes. Activating PI3K/Akt/Nrf2 pathway and Nrf-2 by CA led to the prevention of neurotoxicity caused by glycation (de Oliveira et al., 2015).

#### Others

Seham, M et al. studied the effect of rosemary extract on some neurotransmitters of rats (Na+ ion, GABA contents, monoamines, and acetylcholine esterase) that were reduced significantly in different brain areas of adult albino rats. Rosemary extract demonstrated anticonvulsant and antinociceptive effects by persistent sodium currents blockade in CNS neurons. Moreover, decrement in GABA and enhancement in Cl- ion contents cause sedative and anxiolytic effects of Rosmarinus. It also positively affected rats' memory through anti-acetylcholine esterase (AChE) activity (Seham et al., 2010).

### Lavender

#### Dementia

Lavender has shown efficacy in the treatment of dementia. In 2013, Hancianu et al. conducted a study to assess the neuroprotective effects of inhaled lavender oil on rats with scopolamine-induced dementia. This study and two others showed that a significant part of lavender oil's effects on dementia was exerted through its anti-oxidative and anti-apoptotic properties. Aqueous fraction of L. Stoechas affects dementia in several ways. It reduces AChE which degrades acetylcholine in synapses and can lead to dementia. On the other hand, it reduces neural damage and memory loss due to oxidative stress by elevating the levels of SOD, CAT, and glutathiones. Aqueous fraction can also decrease neuron decay caused by lipid peroxidation by reducing the MDA levels (Hancianu et al., 2013; Mushtaq et al., 2018a,b). Hritcu et al. also studied the effects of lavender essential oil on spatial memory. They impaired the spatial memory of fifty rats using scopolamine, and then, the rats were put in contact with lavender essential oil for 7 days. This study showed that lavender affects the cholinergic system and reverses the memory impairment; therefore, it enhances memory. These effects are mainly due to the presence of linalool in lavender oil (Hritcu et al., 2012). Kashani et al. examined the influences of aqueous extract of lavender on spatial functioning of rats with AD; they understood that lavender extract could excellently improve the spatial learning deficiencies in AD, decreasing glutamate-induced neurotoxicity, leading to neural cell death (Kashani et al., 2011). Zali et al. investigated the specific target proteins in the hippocampus of Aβ syringed rats; the study demonstrated that lavender extract has a positive effect on the performance of AD rats by decreasing Aβ formation. They concluded that lavender extract could be a useful substance as a medication for AD and other kinds of neurodegenerative diseases by expressing neuroprotective proteins in the hippocampus as drug targets (Zali et al., 2015). Soheili et al. demonstrated that aqueous extract of *Lavandula angustifolia* modifies memory deficiencies of AD in animal models. This study showed that the extract of lavender efficiently eliminates Aβ plaques from the brain of rats with AD. The rats were injected with Aβ intracerebroventricularly. The extract reduced the level of Aβ in the brain by plasma clearance of Aβ. Also, lavender can undergo the Aβ's liver clearance because the level of liver clearance of Aβ designated the available levels of plasma Aβ to carry into the brain (Soheili et al., 2012). In another study, Xu et al. evaluated the effect of lavender oil on scopolamine, which is a significant cause of cognitive deficiencies and also on H_2_O_2_ in an *in vivo* and *in vitro* study. The results of this study showed that lavender oil lowered the activity of acetylcholinesterase level of malondialdehyde and increased the activity of superoxide dismutase and glutathione peroxidase. In addition, it could play a role in protection of pc12 cells from H_2_O_2_. It was concluded that lavender oil *in vitro* (H_2_O_2_ in PC12 cells) and *in vivo* (scopolamine mice) could exhibit neuroprotective function, through adjusting acetylcholinesterase function and oxidative stress. The results demonstrated that lavender essential oil treatment restituted the scopolamine induced glutathione peroxidase also superoxide dismutase acting decrease in hippocampus and reduced the high level of malondialdehyde in mice cured by scopolamine. They concluded that this herb had mnemonic effects rather than sensorimotor effects. So based on the correction of cholinergic systems, decreases in oxidative stress could be effective in cognitive problems (Xu et al., 2016).

Pan xu et al. investigated the impacts of lavender essential oil and also its important component, linalool on cognitive disability. This study showed that lavender essential oil and Linalool considerably protected the gained function of acetylcholinesterase and the substance of malondialdehyde also protected the reduced function of superoxide dismutase, glutathione peroxidase. Also, they protected the repressed expression of nuclear factor erythroid 2-related factor 2 and heme oxygenase-1, significantly. Besides, reduced expression of proteins related to synapse plasticity, brain-derived neurotrophic factor, p-CaMKII, and calcium–calmodulin-dependent protein kinase II (CaMKII) was achieved with drug treatment. To sum up, lavender essential oil and linalool could protect nuclear factor erythroid 2-related factor 2 / heme oxygenase-1 pathway protein expression, cholinergic activity, oxidative stress, and synaptic plasticity. Therefore, lavender essential oil and linalool can be considered as a possible agent for the enhancement of cognitive disability in AD (Xu et al., 2017).

#### Glioblastoma

Lavender has also shown efficacy in treating brain tumors. Simsek et al. investigated the antiproliferative and apoptotic inducing impacts of silver nanoparticles and lavender extract combination (La-AgNPs) on human brain tumor cells (U87MG). They concluded that La-AgNPs in the tumor cells can stimulate increased expression of the apoptotic proteins including P53 and caspase 3, −8, and −9. The results of this study show that La-AgNPs can induce apoptosis in these tumor cells by modulating the P53 apoptotic pathway. This study suggested the use of La-AgNPs as a complementary treatment for glioblastoma (Simsek et al., 2021). Chan et al. also did a similar study on apoptotic, cytotoxic, and growth-inhibiting effects of the low-dose lavender extract on two human glioblastoma cell lines (U-87 MG and U-138 MG). They treated cell lines with diluted lavender oil for 2, 4, 8, and 48 h. This study showed that diluted lavender oil could induce apoptosis and cancer cell necrosis. It was reported that the use of lavender essential oil at the concentration of 1:100 has cytotoxic effects through apoptosis and limited necrosis. In this study, they anticipated that an intratumoral shot of lavender will cause the shrinkage of glioblastoma cells by apoptosis mechanism also greater concentrations of this herb, causing a difference between necrotic also apoptotic influences (Chan et al., 2020). On the other hand, Sienkiewicz et al. did a study to compare the cytotoxic effects of cinnamon, geranium, and lavender essential oil. According to this study, lavender oil's cytotoxic effects on glioblastoma cell lines are lower than cinnamon and geranium (Sienkiewicz et al., 2014).

#### Neuroblastoma

Garzoli et al. researched the cytotoxic and antioxidant activities of lavender essential oil and hydrolate on human neuroblastoma cell line SHSY5Y. They exposed this cell line to lavender essential oil and hydrolates for 24, 48, and 72 h. Their study showed that lavender possesses antioxidant abilities (Garzoli et al., 2021). In a similar study, Caputo et al. evaluated the cytotoxic effects of lavender essential oil on human neuroblastoma cell line SHSY5Y. The results show that linalool is the dominant component in lavender essential oil and that linalool is responsible mainly for the cytotoxic effects of lavender essential oil and is highly cytotoxic in 24 h of exposure SHSY5Y cell line. Linalool can have a strong cytotoxic effect due to its ability to damage the cell membrane, cellular attachments, and cell morphology, which can lead to cell death. Since linalool inhibits the expression of adenylyl cyclase and extracellular signal-regulated kinase, it may be the main reason for its cytotoxic effects. Results exhibit that linalool, the basic part of two essential oils, has a more powerful cytotoxic acting vs. SH-SY5Y cells. Also, therapy with various concentrations of linalool reduces adenylate cyclase 1 and extracellular signal-regulated kinase expression (Caputo et al., 2016).

#### Neurotoxicity

Caputo et al. studied the neuroprotective effects of lavender essential oil and its primary active component, linalool, vs. neurotoxicity caused by Aβ1-42 protein components, which have a crucial role in Alzheimer's development, in PC12 cell culture. They exposed these cells to lavender essential oil for 24 h. Their study showed that lavender essential oil could reduce morphological abnormalities and reactive oxygen levels in cells and inhibit proapoptotic enzyme caspase-3 production and function. The lavender essential oil can decrease Aβ1-42 protein components neurotoxicity by neutralizing their impact on Ca ^2+^ homeostasis dysregulation through voltage-gated calcium channels (VGCC) and N-methyl-D-aspartate (NMDA) receptors. Therefore, it prevents this dysregulation from producing reactive oxygen and activating caspase-3, which leads to an apoptotic cascade. They suggested using the lavender essential oil and its main component, linalool, as a potential therapeutic agent for Alzheimer's disease (Caputo et al., 2021). Buyukokuroglu et al. studied the effects of the lavender extract on neurotoxicity caused by glutamate in cerebellar rat cells. They suggested that lavender extract's neuroprotective effect against glutamate-induced neurotoxicity is due to its ability to block calcium channels and anti-glutaminergic activities (Büyükokuroglu et al., 2003). In another study, Soheili et al. evaluated lavender's antioxidant, anti-aggregative, and anti-acetylcholinesterase in an *in vitro* environment. They declared that lavender is not toxic and has antioxidant activities, so it can also be used in treating neurodegenerative diseases such as AD. This herbal drug had no preventive influence on the function of acetylcholinesterase, and also, this drug opposes the fibrillation of Aβ and inhibits the formation of plaque. The lavender extract reduces the extracellular reposition of N-methyl-D-aspartic acid receptors also the neurotoxicity of glutamate (Soheili and Salami, 2019).

#### Epilepsy

Few studies have evaluated lavender's effect on epilepsy. Azimi et al. have studied the impact of the lavender extract on pilocarpine-induced temporal lope epilepsy in rat models. In this study, 75 models were categorized into five groups: saline control, extract control, and epileptic group. The epileptic group received a single dose of lavender extract. Their studies showed that lavender administration delayed status epilepticus occurrence, decreased its duration, and reduced the mortality rate. All these effects may be partly due to higher levels of glutathione. The cells treated with Lavandula dentata extract showed increased levels of glutathione which is a major factor in enhancing cell defenses against oxidative stress. The MDA levels were unchanged (Azimi et al., 2021). In another study, similar results were reported. Sedighnejad et al. researched the effect of the lavender extract on kainic acid-induced TLE in rat models. In this study, 75 rat models were included in five groups: Sham, extract pretreated sham, kainic acid, extract single-dose pretreated kainic acid, and extract repeated pretreated kainic acid. Their results showed that MDA levels were reduced; therefore, number, duration, and mortality rate of SE were reduced (Sedighnejad et al., 2020). Rahmati et al. compared the anti-seizure and antioxidant activities of lavender with valproate in mice in which epilepsy was induced by pentylenetetrazol (PTZ). They concluded that lavender's ability in suppressing nitric oxide (NO) and in reducing malondialdehyde (MDA) was more than valproate. Overall, lavender is more effective in controlling epilepsy (Rahmati et al., 2013). Arzi et al. also studied the efficacy of the lavender hydroalcoholic extract in treating nicotine-induced convulsion in mice. Their study showed that lavender extract has anticonvulsant effects and can increase the onset time and decrease the duration and intensity of convulsion in mice (Arzi et al., 2011). Chaymae et al. evaluated the modulation ability of lavender and rosemary extract on T-type calcium channels (TTCCs). TTCCs play an essential role in epilepsy, pain, sleep, neuroprotection, etc. According to this study, lavender and rosemary exerted an inhibitory effect on TTCCs due to their anxiolytic neuroprotective properties in a dose-related way (El Alaoui et al., 2017). Moheb, Pejhan, Nasab, Nasr also evaluated the anti-inflammatory and anticonvulsant effects of lavender *in vivo*. They induced seizures with pentylenetetrazol (PTZ) in rats. According to their reports, lavender can decrease the severity of a seizure and has neuroprotective effects (Moheb et al.). Mehrabani et al. conducted a similar study and reported that lavender has anticonvulsant effects and can reduce seizure-related mortality rate (Mehrabani et al., 2007). Koutroumanidou et al. also did a similar study on mice and concluded that linalool has analgesic, anticonvulsant, and anti-inflammatory effects. It could also decrease the mortality rate due to seizures and cause a delay in seizure onset (Koutroumanidou et al., 2013).

#### Parkinson's Disease

Nikolova et al. suggested that a mixture of essential oils (Rose and Lavender) and two classic antioxidants such as vitamin C and Trolox with Levadopa (the most efficient drug for Parkinson's) may decrease oxidative toxicity induced by Levadopa and may have an effect on ROS. The anti-oxidative effect was examined by measuring the ranges of 3 biomarkers of oxidative stress, including malondialdehyde, protein carbonyl content, and nitric oxide radicals in blood and brain. The mentioned study showed that all evaluated antioxidants decreased the amount of NO and lipid peroxidation to an equal degree. They also concluded that these herbal essential oils were similar to vitamin c and Trolox in activity (Nikolova et al., 2016).

Ehraz A et al. evaluated the efficiency of perillyl alcohol (PA), which is obtained from essential oils of various plants such as sage, peppermint, and lavender in reducing ROS production and dysfunction of mitochondria and cytotoxicity induced by 6-hydroxy dopamine in Parkinson's disease models. They found that treating with perillyl alcohol relieved the viability loss significantly. perillyl alcohol can enhance Parkinson's disease (PD) by reversing the neurodegenerative effects of 6-hydroxy dopamine such as the production of reactive oxygen species. Also, this drug could increase mitochondrial membrane potential, reduce Cyt c immunofluorescence, and lower tail length and percent tail DNA. They concluded that perillyl alcohol exerts neuroprotective ability and its main role is in restoring mitochondrial membrane potential (Anis et al., 2018). However, not all studies reported the beneficial effects of lavender on Parkinson's disease. Abrishamdar et al. aimed to examine the impact of hydroalcoholic extract of lavender on motor disabilities in Parkinson's disease model rats. Based on this study, lavender extract caused no enhancement in symptoms of Parkinson's disease and motor disabilities. Accordingly, hydroalcoholic extract of lavender had no impact on motor disorders in patients with Parkinson's disease (Abrishamdar et al., 2017).

#### Migraine

Sasannejad et al., in a clinical trial, examined the efficiency of lavender oil inspiration on migraine. There was a remarkable decrease in the intensity of migraine in the first to the sixth attack of headache after the use of lavender in the case group. In the control group, reduction of headache intensity was remarkable after treatment with placebo in the first to the fourth migraine attack, but it was not considered in the fifth to sixth attack. This study concluded that breathing lavender essential oil can be an efficient and safe therapeutic modality in the management of acute migraine headaches. Also, it has been shown that using lavender can develop the risk of recurrent migraine. Lavender is better than oral triptans, acetaminophen, and NSAIDs without many remarkable side effects (Sasannejad et al., 2012). Rafie et al., in a randomized controlled clinical trial evaluated the impact of lavender as a prophylactic treatment for migraine over 3 months. The results showed that lavender exerts a physiological impact on the neural system. Accordingly, the intensity and repetition of migraine attack occurrence were lowered in participants consuming lavender therapy. Linalool and linalyl acetate which are the basic compounds of this drug had positive effects on the nervous system. Also, it had sedative impressions (Rafie et al., 2016). Jafari-Koulaee et al. evaluated the impact of aromatherapy with lavender on dumps and disabilities of headache in a clinical trial. The severity of depression and disabilities of headaches were evaluated by Beck Depression Inventory and Jacobson questionnaire. The results of the study showed that in the experimental group, scores of depression and headache disability were completely different in the period before the intervention, 2 and 4 weeks after aromatherapy between the two groups, no difference was found in terms of disabilities of headache and depression scores during mentioned periods. Accordingly, lavender essential oil may be efficient for decreasing disabilities of headache and depression in patients with migraine (Jafari-Koulaee et al., 2019).

#### Multiple Sclerosis

Multiple sclerosis (MS) is a disabling disease of the brain and spinal cord. In MS, the immune system attacks the protective sheath that covers nerve fibers (Dobson and Giovannoni, 2019). Fard et al. evaluated the influence of Lavandula essential oil upon brain-derived neurotrophic factor and interleukin-23 gene expressions in peripheral blood mononuclear cells of patients with relapse remitting MS. This study demonstrated that Lavandula essential oil could have a protective impression against neuron destruction by raising the gene expression of brain-derived neurotrophic factors in patients with relapse remitting patients with MS in peripheral blood mononuclear cells (Fard et al.). Seddighi-Khavidaka et al. evaluated the influence of lavender oil as an olfactory motivation and vestibular rehabilitation on the balance and function of daily living of patients with MS. The group who was treated with lavender had a better performance in the tests: the 29-item MS impact scale, Berg balance scale, Timed Up and Go TEST, and fall efficacy scale—international compared to the control group. The study reported that lavender oil as an olfactory motivation has a beneficial effect on minimizing concern of falling down and balance during doing the vestibular rehabilitation exercises compared to not using it in patients with MS by stimulating the insular cortex (Seddighi-Khavidak et al., 2020). In another study, Majidinasab et al. evaluated the impact of Iranian Lavandula on trembles or tremors in patients with MS. In this randomized, double-blind clinical trial, the Wilcoxon test showed a considerable difference in the tremor of the group treated with lavender. To sum up, this herbal medicine with the least amount of 80 mg is efficient in decreasing the tremor in patients with MS, and this drug can be consumed alone or as a secondary drug (Mehravar, 2020).

#### Others

In some neurologic diseases such as stroke, epilepsy, ALS, Alzheimer's, and so on., α-amino-3-hydroxy5-methyl-4-isoxazole propionic acid receptor (AMPAR) becomes overactivated. Qneibi et al. studied the effects of lavender essential oil on AMPAR activity and showed that lavender essential oil modifies its activity. Lavender acts as an antagonist to the AMPA receptor and prevents the glutamate from bonding to it and consequently prevents Ca2+ dysregulation and the synaptic dysfunction in these diseases. They concluded that lavender essential oil could be used as an antagonist for AMPAR and a neuroprotective drug for the so-called neurologic diseases (Qneibi et al., 2019).

### Sage

#### Cognitive Deficits

The effect of sage has been studied on cognitive deficits. Kennedy et al. studied 36 healthy subjects receiving oral sage extract. These results showed improvement in secondary memory performance, decreased mental fatigue, and elevated alertness by inhibition of AChE (Kennedy et al., 2011). In 2018, Wightman EL et al. examined the effects of the sage combination on cognitive performance in 94 individuals. The considerable benefit of this combination was observed in cognitive accuracy and working memory both acutely and chronically. Probably, a large adjustment of the CBF response is responsible for the significant increase in cognitive performance after consuming Greek mountain tea on Day 1. The benefits on Day 28 are more likely to be related to reduced state anxiety, implying that the former mechanism is more likely to facilitate acute cognitive impacts and the latter mechanism is more likely to underpin long-term cognitive improvements (Wightman et al., 2018). A total of two other studies (on young adults and adolescents) were conducted with a single dose of 150 and 300 mg of sage extract, respectively. A marked improvement following the sage extract consumption was shown for evaluating cognitive performance and short-term memory in healthy young adults, which are similar to previous studies in older adults (Edwards et al., 2021). Similarly, a recent RCT in young and healthy cases demonstrated the combination of various phenolic and aromatic extracts such as sage might have significant beneficial effects on the function of the brain (Jackson et al., 2020). Kennedy et al. administrated different concentrations of sage (300, 600 mg dried sage leaf, placebo) in 30 healthy young individuals, leading to improvement in cognitive performance, mood, and anxiety state by cholinesterase inhibiting effects of sage (Kennedy et al., 2006). In 2019, Tober C et al. considered a wide pharmacodynamic spectrum of *Salvia Officinalis* with special effects, seen on serotonin neurotransmission, as well as on μ-opioid, muscarinic M3, and adrenergic α2A receptors. They suggested powerful modulation of serotonin transporters and neuroreceptors as a manner of action, which may normalize thermoregulatory responses and possibly mental impairment before menopause. Also, using fresh *Salvia officinalis* leaves had higher activity than extracts from dried plants (Tober and Schoop, 2019).

#### Dementia

Akhondzadeh et al. investigated the effect of *Salvia officinalis* extracts on the treatment of mild to moderate cases of AD. They designed a randomized trial with 39 patients in Tehran. Mild to moderate cases of AD aged 65–80 years were included to take certain doses of *Salvia officinalis* extract or placebo. After 4 months, *Salvia officinalis* extract showed a considerably better effect on cognitive functions compared to placebo (Akhondzadeh et al., 2003). Abozaid et al. evaluated the possible effect of Mg-*Salvia officinalis* nanoparticles on the treatment of aluminum chloride (ALCL3) induced AD in rats. The study reported that ALCL3-induced AD in rats causes considerable changes in acetylcholine levels, and tau protein in brain tissue. Treatment with *Mg-salvia officinalis* nanoparticles in AD rats caused significant improvement in these parameters. In conclusion, *Mg-salvia officinalis* nanoparticle treatment improves oxidative stress and boosts the antioxidant defense system (Elkomy et al., 2021). Fatima et al. investigated the neuromodulatory effects of the sage extract on aluminum chloride-intoxicated rats. They found that administrating 500 mg/kg of this extract not only improves behavioral parameters but also reverses the decreased acetylcholinesterase content. Thus, the advantageous effects of sage in alleviating Alzheimer's disease were demonstrated due to its acetylcholinesterase inhibiting and antioxidant activity and its ability to improve cognitive functions and memory (Fatima and Tabassum, 2020).

Scholey et al. performed a randomized, double-blind, placebo-controlled study to evaluate the effects of an extract of *Salvia officinalis* on cognitive performance in elderlies. A total of twenty participants aged >65 received a placebo and four doses of *Salvia officinalis* extract (167, 333, 666, and 1,332 mg). They concluded that the 333-mg dose was associated with a remarkable improvement in secondary memory. The other doses showed the same effect to a lesser extent. In addition, the extract inhibited anti-cholinesterase activity, resulting in a 50% reduction in substrate breakdown by human cholinesterase (Scholey et al., 2008). However, not all studies reported the beneficial effects of sage on memory. Perry et al. evaluated the effects of a combination of sage, melissa, and rosemary on verbal recall in healthy participants. A total number of 44 healthy subjects participated in this double-blind, randomized, placebo-controlled pilot study. Overall, no significant differences were observed between treatment and placebo groups from the baseline for immediate and delayed word recall. Although, subgroup analysis illustrated those participants under 63 years old showed considerable improvements in delayed word recall, and no adverse effects were seen (Perry et al., 2018).

#### Glioblastoma

Choukairi et al. investigated the effects of *Salvia officinalis* (sage) and *Rosmarinus officinalis* (rosemary) extracts on human GBM cell lines. The study showed that the extracts of *Salvia officinalis* and *Rosmarinus officinalis* remarkably decreased the rate of cell proliferation and showed a dose-dependent antioxidant activity causing an antitumoral effect on GBM (Choukairi et al., 2020).

#### Neurotoxicity

Iuvone et al. evaluated the effects of *Salvia officinalis* and rosmarinic acid extracts on AD Aβ peptide-induced toxicity. Cultured rat pheochromocytoma cells were used to perform the study. Cultured rat pheochromocytoma cells were incubated with AD Aβ for 24 h and it caused cell death. This effect was decreased by *Salvia officinalis* and rosmarinic acid. It was concluded that sage has a neuroprotective effect against AD Aβ induced toxicity. A possible set of events that leads to cell death, based on the current findings and those discovered in the literature, could be as follows: Rosmarinic acid suppresses the generation of ROS and thereby lipid membrane peroxidation, inhibiting Aβ-dependent oxidative stress neurotoxicity and death. Furthermore, rosmarinic acid may block p-p38 MAP kinase, which hyperphosphorylates tau protein, inhibiting the formation of NFT, by lowering ROS production during the early phase of Aβ insult, suggesting that the traditional usage of this spice in the treatment of may have a pharmacological foundation (Iuvone et al., 2006). Boussadia et al. evaluated aqueous *Salvia officinalis* extract effects on aluminum chloride-induced neurotoxicity in female rats. Long-term aluminum chloride exposure caused behavioral deficits by decreasing locomotor activity. Also, it caused a considerable decrease in spontaneous alternation. Furthermore, aluminum chloride exposure demonstrated a significant decrease in acetylcholinesterase and catalase activity. It significantly increased the mean concentration of malondialdehyde compared to the control group. This study showed that *Salvia officinalis* could be administered to protect against the neurotoxicity of aluminum chloride and to improve behavioral and oxidative status, by inhibiting lipid peroxidative malondialdehyde product in their brains and enhancing catalase activity. Antioxidant compounds found in *Salvia officinalis* can provoke endogenous antioxidant defense systems as well as trapping reactive species (Amina et al., 2020).

#### Oxidative Stress

Osman et al. conducted a study on the possible effects of sage on alleviating adverse impacts of gamma radiation-induced oxidative stress on rats' brains. The extract of *Salvia officinalis* was consumed by experimental rats 14 days before and 14 days after their exposure to gamma radiation. They discovered that sage extract has the ability to remarkably raise the level of thiobarbituric acid reactive substances, nitric oxide content, and protein carbonyl content in irradiated rats' brains. Moreover, activities of brain acetylcholinesterase, acid phosphatase, alkaline phosphatase, and lactate dehydrogenase were increased considerably. Conversely, the activity of superoxide dismutase and catalase was diminished. Protecting cell viability, which has been attributed to *Salvia officinalis* extract may be due to its ability to prevent glutathione depletion by its main phenolic compounds. Therefore, it can increase glutathione levels, probably by inducing glutathione synthesis. Therefore, it was concluded that antioxidant constituents of sage extract could contribute to its modulatory effect on oxidative stress induced by radiation, and also on the activities of the brain's enzymes (Sh, 2013). Shahmohamadi et al. examined the effects of hydroalcoholic extract of sage on protection against oxidative stress, by measuring the activity of superoxide dismutase in addition to catalase on male rats receiving an intracerebroventricular injection of streptozotocin. About 25, 50, and 100 mg/kg of this extract were given to the rats intraperitoneally before streptozotocin exposure. As a result, the levels of superoxide dismutase and catalase elevated noticeably, indicating a potential for preventing oxidative stress in rats' brains (Shahmohamadi et al., 2013).

In another study, El-Habibi et al. investigated the potential role of ethanolic extract, water extract, and oil of sage in alleviating brain disturbances caused by aluminum exposure in rats. Their results confirmed that various forms of sage could be capable of antagonizing aluminum-induced cerebral perturbation of several neurotransmitters, due to the antioxidant activities they have. Therefore, it may be used as a neuroprotective compound through regulating neurotransmission and other functions of aluminum intoxicated brains (Taher, 2011). Lipid peroxidation is a series of reactions that results in oxidative degradation of lipids. In this process, free radicals take electrons from the lipids of cell membranes, contributing to cell damage (Santanam et al., 1998). Oboh et al. examined the influence of *Salvia officinalis* leaf's aqueous extract on lipid peroxidation induced by prooxidants in rat brains. In this study, the content of acid ascorbic, Fe (II) chelating, total phenol, and hydroxyl radical scavenging ability of sage extract was evaluated as indices representing antioxidant activity. This study found that this extract has high contents of all the cited indices above, except for hydroxyl radical scavenging ability. Furthermore, it was revealed that the production of malondialdehyde is inhibited by this extract. Therefore, it was concluded that since this leaf has antioxidant and protective properties, it can be utilized for managing and preventing degenerative diseases caused by oxidative stress, especially in the brain and liver (Oboh and Henle, 2009).

#### Ischemic Stroke

Ischemic stroke happens when an artery supplying blood flow to the brain is blocked. Vasogenic edema is an important part of ischemic stroke through the breakdown of the blood–brain barrier (BBB) (Doyle et al., 2008). Seyedemadi et al. evaluated the abilities of hydroalcoholic extract of sage concerning ischemic tolerance in rats. They discovered that pretreating brain ischemia with this extract could remarkably decrease infarct volumes of cortex and sub-cortex, cerebral edema, BBB permeability, and neurologic deficit scores, compared to the control group. Thus, sage hydroalcoholic extract, if used as a pretreatment, can play a crucial role in augmenting tolerance against cerebral injuries (Seyedemadi et al., 2016). Similarly, Ghasemloo et al. investigated the impact of hydroalcoholic extract of sage on the permeability of the BBB, as well as neurological deficits, in transient ischemic model rats. They discovered that administration of 50, 75, and 100 mg/kg of this extract decreased the BBB permeability and the latter two doses diminished neurological deficits in comparison with the control group. Therefore, this extract could have the potential to guard the brain against ischemia (Ghasemloo et al., 2016).

#### Multiple Sclerosis

The effect of Sage on MS has also been studied. Li et al. investigated the effects of carnosol (a compound that originated from *Rosmarinus officinalis* and *Salvia officinalis*) in experimental autoimmune encephalomyelitis (an animal model of MS). The results showed that carnosol treatment considerably reduces demyelination and inflammation in the CNS and alleviates clinical development in experimental autoimmune encephalomyelitis by inhibiting T helper 17 cells (proinflammatory cells) polarization and decreasing inflammatory cell infiltration into the CNS. They concluded that carnosol has great potential for the treatment of autoimmune diseases such as MS (Li et al., 2018). It has been shown that decreased sex hormones may modulate brain damage in MS (Tomassini et al., 2005). Khajehei et al. examined the effect of *Salvia officinalis* on the levels of the hormones LH, FSH, and testosterone in mice with MS. Experimental results showed that LH, FSH, and testosterone were increased in mice consuming sage compared to the control group (Khajehee and Tadjalli, 2017).

#### Seizure

Namvaran-Abbasabad et al. investigated the effects of *Salvia officinalis* hydroalcoholic extract on pentylenetetrazole-induced seizure threshold in vincristine injected mice. The results showed that hydroalcoholic extract of *Salvia officinalis* considerably increased the seizure threshold, so the extract can be used to reduce vincristine-induced neuropathic effects (Namvaran-Abbasabad and Tavakkoli-Ghazani, 2012). Khayate-Nouri et al. evaluated the effects of *Salvia officinalis* and Cisplatin on pentylenetetrazole-induced seizure threshold in mice. The results showed that *Salvia officinalis* extract can decrease cisplatin-induced epileptic and neuropathic effects *in vivo*. Pentylenetetrazole acts picrotoxin action complex of GABA receptor. This study determined that *Salvia officinalis* increased the threshold of pentylenetetrazole-induced seizures. Because pentylenetetrazole acts through GABA receptors, one anticonvulsant effect of Salvia officinalis is likely *via* the GABAergic system. *Salvia officinalis* contains apigenin that has GABA-like effects. Thus, it can augment GABA effects on these receptors. Since GABA is a kind of inhibitory neurotransmitter in the brain, it diminishes activity in CNS (Khayate-Nouri et al., 2013). In another study, Heydari et al. evaluated the effects of hydroalcoholic extract of *Salvia officinalis* on pentylenetetrazol-induced seizure threshold in mice. However, in this study, hydroalcoholic extract of *Salvia officinalis* did not seem to have an anticonvulsant effect (Heydari et al., 2013).

#### Others

Ghasemloo et al. conducted effects of *Salvia officinalis* on neuromotor deficits and BBB permeability in the transient ischemic rat model. They indicated that *Salvia officinalis* could reduce neurologic deficits and permeability of blood–brain barrier (Ghasemloo et al., 2016).

## Conclusion

Altogether, the included articles studied the effects of aromatic herbs, lavender, rosemary, and sage, extracts on cognitive disorders, Alzheimer's, dementia, epilepsy, seizure, glioblastoma, glioma, migraine, neuroblastoma, Parkinson's, and MS. The aromatic herbs were effective in several neurological diseases, and this review suggests these as potential complementary medicines. This review shows the potential effect of rosemary extract on improving cognitive activity, the regulation of mood disorders through increasing the concentration of BDNF and reducing discrete mental stress in elderly people; also, in experimental studies, it showed powerful antioxidant, anti-inflammatory, anti-acetylcholine esterase activities, as well as neuroprotective effects. The anti-cancer effect of rosemary in neuroblastoma cells is exerted by inducing apoptosis through ROS, MAPK, and p38 phosphorylation. Also, rosemary promotes ERK1/2 and decreases cell death in Parkinson's and reduces agitation state in patients with dementia. The animal model studies demonstrated that lavender extract has a potential effect on performance in AD Aβ formation. Also, the lavender essential oil can affect cognitive problems by decreasing oxidative stress based on cholinergic systems. Some research showed that lavender acts as an antagonist to the AMPA receptor and prevents the glutamate from bonding to it and consequently prevents Ca2+ dysregulation and the synaptic dysfunction in these diseases. This study demonstrated that Lavandula essential oil could have a protective impression against neuron destruction by raising the gene expression of brain-derived neurotrophic factors in patients with relapse remitting patients with MS in peripheral blood. Clinical trial studies investigated the effect of sage on AD and dementia through the improvement in secondary memory and cognitive functions. In animal models of seizure and MS, the noticeable enhancement of seizure threshold and reduction of demyelination and inflammation in the central nervous system were observed, respectively. In addition, the extract can affect dementia by inhibiting anti-cholinesterase activity, resulting in a 50% reduction in substrate breakdown by human cholinesterase. Generally, evidence supports these aromatic herbal extracts in various neurological diseases decreased the level of oxidative stress and inflammation markers. In the future, more clinical trials are required to investigate the effects of aromatic herbal medicines for the management of neurological disorders, and more *in vivo* and *in vitro* studies are needed to better identify the underlying mechanisms of action of these herbal medicines in different diseases.

## Author Contributions

ND designed, supervised, and critically revised the manuscript. MS and HS revised the manuscript. AF, YS, HG, MK, AR, GM, MAK, NZ, NJ, AM, and BSD drafted the manuscript. AF did the data collection. All authors contributed to the article and approved the submitted version.

## Conflict of Interest

The authors declare that the research was conducted in the absence of any commercial or financial relationships that could be construed as a potential conflict of interest.

## Publisher's Note

All claims expressed in this article are solely those of the authors and do not necessarily represent those of their affiliated organizations, or those of the publisher, the editors and the reviewers. Any product that may be evaluated in this article, or claim that may be made by its manufacturer, is not guaranteed or endorsed by the publisher.
